# I (Don’t) want to consume counterfeit medicines: exploratory study on the antecedents of consumer attitudes toward counterfeit medicines

**DOI:** 10.1186/s12889-022-13529-7

**Published:** 2022-06-01

**Authors:** Sylvester Senyo Ofori-Parku, Sung Eun Park

**Affiliations:** 1 School of Journalism and Communication, University of Oregon, Eugene, OR USA; 2 School of Communication, Webster University, St. Louis, USA

**Keywords:** Counterfeit medicines, Substandard medicines, Consumer attitudes, Risk perception, Purchase intentions, Pharmaceutical industry, Subjective norms

## Abstract

**Background:**

Substandard and falsified medicine (SFM) sales (an estimated > $200 billion) has become one of the worlds’ fastest growing criminal enterprises. It presents an enormous public health and safety challenge. While the developed world is not precluded from this challenge, studies focus on low-income countries. They emphasize supply chain processes, technological, and legal mechanisms, paying less attention to consumer judgment and decision-making aspects.

**Methods:**

With attention to the demand side of the counterfeit medicines challenge, this survey of U.S. consumers (*n* = 427) sheds light on some of the social, psychological, and normative factors that underlie consumers’ attitudes, risk perceptions, and purchase intentions.

**Results:**

Consumers who (a) self-report that they know about the problem, (b) are older, (c) view counterfeit medicine consumption as ethical, and (d) think their significant others would approve of them using such products are more inclined to perceive lower risks and have favorable purchase intentions. Risk averseness is also inversely related to the predicted outcomes.

Perceived benefit of SFMs is a factor but has no effect when risk perception and aversion, attitudes, and subjective norms are factored into the model that predicts purchase intentions.

**Conclusion:**

The results of this study indicate that consumer knowledge (albeit in an unexpected direction), people’s expectations about what will impress their significant others, their ethical judgments about selling and consuming counterfeits, and their risk-aversion are associated with their decision-making about counterfeit medicines. The study offers insights into a demand-side approach to addressing SFM consumption in the U.S. Implications for public health, consumer safety, and brand advocacy education are discussed.

**Supplementary Information:**

The online version contains supplementary material available at 10.1186/s12889-022-13529-7.

## Introduction

The illicit trafficking and consumption of fake and substandard medicines has become one of the worlds’ fastest growing criminal enterprises during the past two decades globally [1–4]. This phenomenon is fueled by factors such as the lack of access to medical care, consumers’ appetite for cheap medicines, corruption in governments, the proliferation of illicit online pharmacies, the complexity of medical product supply chains, and the availability of sophisticated technologies for counterfeiting and packaging products [1–3, 5, 6]. Although often framed as a third-world problem [7, 8], the challenge is not limited to the developing world. According to estimates, between 10 to 60% of the drugs distributed in the developing world and the vast majority of those sold online in the U.S. are considered “counterfeit” [9, 10]. Also, Rahman et al. [11] found that out of 48 recorded incidences of health impairment owing to fake medicines, they were virtually evenly split between developing (27 cases, 56.3 percent) and developed countries (21 cases, 43.7 percent). This study focuses on the demand side of the issue. It assesses some social, psychological, and normative determinants of consumer attitudes and intentions to patronize such medicines in a developed country context: United States.

Quantifying the global counterfeit medicines market is exceedingly difficult. For example, the Organization for Economic Co-operation and Development (OECD) pegs the size of the *international* trade (based solely on customs seizure statistics) in counterfeit medicines at $4.4 billion in 2016 [2]. As OECD’s 2020 report explains, this figure “does not include a very large volume of domestically produced and consumed illicit pharmaceuticals” ([2] p. 11). Other analysts estimate “counterfeit” medicine *overall sales* to be worth between $200 billion [3, 12] and $432 billion annually [13]. Miller and Winegarden’s [12] sales estimate make fake medicines the number one illegal goods (in terms of sales), ahead of other illicit trafficking activities such as prostitution and marijuana. The OECD (2020) data also identifies counterfeit pharmaceuticals as a top 10 (out of 97) recorded product categories based on customs seizures [2].

Generally, counterfeit medicines raise brand equity and brand safety concerns [4], leading to over $80 billion in financial loss each year [2, 14]. However, this research focuses not on the brand equity, intellectual property, and competitive advantage implications of “counterfeits medicines” as a catch-all phrase but on the health and safety risks of fake pharmaceutical products. There is no universally accepted definition of “counterfeit medicines.” The World Health Organization (WHO) originally used the term “substandard, spurious, falsely labeled, falsified, and counterfeits (SSFFC) to describe these medical products. Substandard medical products are often designed to appear identical to genuine product and may not cause an obvious adverse reaction [15]. However, such medications often fail to properly treat the disease or condition for which they were intended, and can lead to serious health consequences including death [15]. Falsified drugs “deliberately/fraudulently misrepresent their identity, composition or source” ([15] para, 8). A recent systemic review of 47 global studies on medicine quality studies, McManus and Naughton [8] identified the following categories of issues and their prevalence rates: inadequate amount of active ingredients (94%), dissolution failure (39%), no active ingredient (18%), excessive amount of active ingredients (12%), wrong ingredients (3%), and impurities (3%).

In line with this, “counterfeit medicine” is used narrowly in this study to mean “substandard and falsified medicines” (SFMs) [2, 8]. The SFM terminology emphasizes the threat to public health and safety, not intellectual property infringements of illegally “copying” original pharmaceuticals as “counterfeit” connotes [2, 15]. Specifically, the term refers to “falsified medicines” that are fraudulently produced and distributed, do not meet quality specifications, but are sold “with the explicit intent to deceive the end-user of their origin, authenticity, and efficacy” ([8] p. 1). It also entails “substandard drugs” that do not have the right or correct amounts of active pharmaceutical ingredients. The term as used here is not synonymous with low-cost generics that are as safe and effective as existing brand-name versions protected by intellectual property [15]. For example, such low-cost copies of medicines (that are not substandard) have proved to be life-saving, cheaper alternatives for fighting health problems (see Ghinea et al. [5] for debate on medication pricing and low-cost generic importation regulations). Besides, while, in theory, fake medicines that infringe on the copyrights of innovator brands may contain the right kind and quantities of active ingredients, enforcement and industry experts explain that such cases are virtually non-existent [2].

All types of medications have been falsified [11]. They include generics and “innovator” ones; life-saving drugs for illnesses such as cancer and those for routine ailments such as painkillers; antimalarials; antibiotics; and cheap as well as expensive drugs. The internet is playing an increased role in the proliferation and consumption of substandard and falsified medicines [2, 10]. The European Alliance for Access to Safe Medicines (EAASM) found that over 90% of websites that sell medications did not require prescriptions, and 62% of the medicines sold on these websites were falsified or substandard [16]. Only four percent of randomly sampled online pharmacies (out of 11,700) adhere to U.S. pharmacy laws and practice standards [17].

A recent study on online no-prescription somatropin medicines [18] found results similar to EAASM: most (94%) did not require valid prescriptions and were substandard. Further, all online medication samples analyzed contained significantly lower active ingredient concentrations than labeled. All of this notwithstanding, “nearly one in four adult consumers has purchased prescription medicines online and almost one in five of [of them] bought from a website that was not associated with a local pharmacy or health insurance plan” in the U.S. ([19] para 8). Generally, consumers who frequently buy online and spend more time on the internet have more favorable attitudes toward online pharmacies than those who do not [20]. (The focus of this study is, however, not on where SFMs are accessed or sold. Thus far, the discussion is to illustrate and reflect on how easy it is to access substandard and falsified medicines.)

Besides their implications for pharmaceutical brands, SFMs proliferation is a more significant public health threat than diseases they purport to cure [8, 21]. They have dire long-term health consequences for consumers (e.g., organ failure, antimicrobial resistance, overdose, or even death) [6, 8, 10, 15]. As Lybecker [21] observes, counterfeiting is a less understood, invisible barrier to medication access and safety compared to pharmaceutical pricing. Thus, medication access does entail not only availability and affordability but also quality [22]—all three of which relate to SFMs. The health, safety, risks notwithstanding, most people, including Americans, are unaware of the prevalence of the problem and the consequences of purchasing and taking such drugs [2, 4, 20, 21]. The lack of rigorous and universal drug regulatory frameworks, the complexity of drug supply chains and the sophistication of medicine packaging make it difficult for regulators, pharmaceutical firms, activists, and consumers to detect counterfeit drugs [6]. Much of the fake medicine problem comes from the globalization of the pharmaceutical industry itself [2, 14, 18]. With an eye on cost reduction and competitiveness, many companies have outsourced the supply of ingredients and even the actual manufacturing of their final goods around the globe (e.g., China and India).

The falsified and substandard medicines problem straddles business and public health, given the public health and safety, financial, and brand equity implications [6, 10, 23]. This study was part of a larger project on SFMs as global health, brand, marketing, and public policy challenge. It examines the association between demographic factors (i.e., age and income), self-reported knowledge of the problem, ethical judgment, risk aversion and subjective norms (on the one hand), and consumers’ attitudes toward falsified and substandard medicines, their risk perception, and purchase intentions (on the other hand). Despite the pervasiveness of the substandard and falsified medicines challenge, existing research (except for a few studies in low-income countries [7, 21]) has mainly focused on the supply chain. Others concentrate on regulatory conditions and technologies that make it challenging to—or can help—address the challenge [14, 24]. Pharmaceuticals are increasingly adopting technologies to support electronic tracking or point of purchase verification codes (e.g., mPedigree). But some manufacturers claim such technologies are unreliable and increase drug costs [24]. Wechsler [24] also observes how pharmacists protest taking on the additional responsibility of checking the authenticity of every drug coming in from wholesalers and distributors. Besides, the pharmaceutical industry insists that counterfeit detection and resistance technologies must be regularly rotated as counterfeiters can easily duplicate them within 12–18 months [14]. These observations suggest the importance of a complementary consumer-facing, demand-side approach, which considers the socio-cognitive antecedents of consumers’ judgment and decision making. The decision-making process is further complicated by packaging characteristics not being reliable markers of authenticity [25] since counterfeits and genuine drugs tend to look identical. Complementing studies on how policymakers can curtail the SFM market to ensure health and safety, we focus on the consumer. Understanding the psycho-social factors that underlie their attitudes and purchase intentions can inform public health communication and advocacy efforts to improve consumer decision-making.

### Literature and hypotheses

Given the lack of theoretical development on consumer attitudes toward SFMs, this study set out to ascertain some predictors of consumers’ attitudes toward falsified medicines (to know how best to engage them). The study is based on aspects of the theory of planned behavior and reasoned action [26, 27] and literature on consumer behavior in general consumption contexts and risk perception and decision-making. We propose six hypotheses and three research questions. Each hypothesis (except H1) had three dependent variables: attitudes toward SFMs, risk perception, and purchase intent.

While the global falsified and substandard medicines challenge transcends legal, regulatory, and engineering considerations, studies examining this problem are limited in scope, often framing the problem in terms of low-resource countries (see systematic review by McManus and Naughton [8]). In response to this, some researchers have long suggested that communication strategies need to be implemented to address the safety issue of using SFMs and traits that consumers can use to detect counterfeits [28]. The study developed partly in response to these calls to execute aggressive campaigns to increase public awareness of counterfeits [29–31], implement anti-counterfeit programs that emphasize the quality and safety of using authentic products, and develop tailored communication strategies to address attitudes and beliefs about counterfeits [32].

To deliver compelling messages about fake drugs and increase public awareness, advocates’ understanding of the motivations or predictors of using counterfeits is essential. For example, Nigeria spent over $68 million trying to address the fake medicines challenge over a decade ago but has made little progress [25]. Given the lack of studies on consumer attitudes toward substandard and falsified medicines in general and the United States, we observe some lessons from the few studies in low-income countries. The study also borrows from the literature on consumer behavior regarding counterfeit products in general consumer contexts (although counterfeited medicines are, arguably, different from other consumer goods). These studies suggest that social norms, demographics, perceived risks, risk aversion, and ethical judgment are associated with consumer attitudes and purchase intentions toward counterfeit products [7, 21, 33–41]. In non-pharmaceutical contexts, perceived risk, whether individuals view consuming such products as fair or unfair, and whether they feel counterfeit products make a positive contribution to their well-being is associated with consumer attitudes and purchase intentions [39]. The association between perceived risk and consumer attitudes is such that individuals who view counterfeit products as risky are less likely to consume counterfeit products [34, 42–45]. Besides, when people think the social costs victims of counterfeit products incur are too high, they disapprove of fake products [36]. Thus, we hypothesize that:

- **H1a**: There is an inverse relationship between the risk consumers associate with SFMs and their attitude toward such medication.
- **H1b**: Consumers’ perceived risk of SFMs is negatively associated with their purchase intentions.

Overall, people’s ethical judgments about counterfeit medications are associated with their attitudes, consumption intentions, and behaviors. Those who see buying counterfeit consumer products as unfair or unethical tend to have unfavorable attitudes and purchase intentions [35, 38, 39, 45, 46]. Hence, we hypothesized that:

- **H2a**: The ethical judgments consumers make about SFMs have a negative effect on their overall attitude toward such medicines.
- **H2b**: There is a positive relationship between consumers’ ethical judgment about SFMs and how much risk they associate with such medication.
- **H2c**: There is a negative relationship between consumers’ ethical judgment about SFMs and their purchase intentions.

Studies in non-pharmaceuticals contexts [34, 35, 42, 46] also suggest that consumers who have bought counterfeit products in the past have more favorable views on such products. Thus, knowing about or having experience with counterfeit products may not necessarily be associated with unfavorable attitudes toward such products. Our third set of hypotheses predicted that:

- **H3a:** Consumers’ self-reported knowledge of SFMs is inversely related to their attitudes toward such medicines.
- **H3b:** Consumers’ self-reported knowledge of SFMs positively correlates with the risk they associate with SFMs.
- **H3c:** Consumers’ self-reported knowledge of SFMs is inversely related to their intention to purchase such drugs.

Further, as the theory of planned behavior and reasoned action propose, individuals’ subjective norms [26, 27] have implications for their attitudes, intentions, and behaviors. This mechanism is also termed normative susceptibility —people taking actions based on their expectations about what will impress others [7, 27, 39]. In simple terms, subjective norms refer to individuals’ perception or “opinion about what important others believe the individual should do [or not do in a specific situation]” ([47] p. 2015]). Applied to counterfeit products, extant research [7, 39, 46, 48] shows that when consumers think people who are important to them (e.g., family and friends) will disapprove of their decision to patronize counterfeit products, they tend to have unfavorable attitudes and purchase intentions. Therefore, the fourth hypothesis predicted that:

- **H4a**: There is a positive relationship between consumers’ subjective norms and their attitudes toward consuming SFMs.
- **H4b**: There is a negative relationship between consumers’ subjective norms and risk perception.
- **H4c**: There is a positive relationship between consumers’ subjective norms and purchase intentions.

Further, research on counterfeit products in general consumption contexts links risk aversion to consumer attitudes toward and intention to purchase such products. Individuals with a predisposition to avoid risks tend to express concern over the efficacy of counterfeit products and how safe they are [39, 44, 46]. Similar to the effect of risk perception on consumer attitudes toward counterfeit products [34, 42], risk aversion can negatively affect consumers’ attitude toward counterfeit goods [44]. In line with these studies, our fifth set of hypotheses predicted that:

- **H5a**: Risk aversion is negatively related to attitude toward purchasing SFMs.
- **H5b**: There is a positive relationship between risk aversion and consumers’ risk associated with SFM consumption.
- **H5c**: There is an inverse relationship between risk aversion and consumers’ risk associated with SFM consumption.

Regarding demographics, some studies suggest that income is not a significant determinant of consumers’ intention to purchase counterfeits (e.g., [42, 49]). But others have associated having lower income levels and being young with favorable attitudes toward counterfeit goods [39, 41]. It is reasonable to expect that people of lower socioeconomic status are most likely to patronize SFMs because of price incentives or economic concerns. This may not always be the case, however. For example, individuals who order medications —often SSFFCs— from no-prescription websites tend to be literate and have relatively high socioeconomic status [50, 51]. Although price incentives are often cited as a reason for online medication purchases (94% of which tend to be fake), for some medications, SFM online versions can be more expensive (40–65% higher) than genuine brands [18]. The mixed results on income and SFM purchase intentions notwithstanding since counterfeit medicines tend to be, perceived as, or marketed as cheaper [2, 18], we hypothesize that:

- **H6a:** There is an inverse relationship between consumers' income and their attitude toward SFMs.
- **H6b**: There is an inverse relationship between consumers’ income and the perceived risks of SFMs.
- **H6c**: Consumers who earn more are less likely to purchase SFMs than those who earn more.

As Tom et al. [41] found concerning age, individuals who have purchased counterfeit products in the past are “significantly younger” than those who have never purchased faked goods. But studies linking age and consumer behavior relating to counterfeits are inconclusive. For example, other researchers [42, 49] have found no significant relationship between the two variables. Therefore, we pose no specific hypotheses; instead, our first research question asked:

- **RQ1a**: To what extent does attitude toward counterfeit drugs differ by age?
- **RQ1b**: To what extent does risk perception differ by age?
- **RQ1c**: To what extent does purchase intention for counterfeit drugs differ by age?

The second set of research questions addresses the cumulative relationship between our predictor variables of interest and the specified outcomes.

- **RQ2a**: Controlling for age, to what extent do consumer knowledge, ethical judgment, risk aversion, and subjective norm predict their overall attitudes toward SFMs?
- **RQ2b**: Controlling for age, how do consumer knowledge, ethical judgment, risk aversion, and subjective norm predict their overall risk perception?
- **RQ2c**: Controlling for age, to what extent do consumer knowledge, ethical judgment, risk aversion, and subjective norm predict consumers’ purchase intentions?

## Method

### Participants

The researchers collected 427 valid samples through Amazon’s Mechanical Turk (MTurk), a crowdsourcing service. Social science experiments and surveys are increasingly using MTurk samples [52–54]. Despite these samples being self-selected, they are representative of the general United States population on characteristics such as party identification, political ideology, geographical categories, education, age, marital status, religion, and employment than in-person convenience samples [55, 56].

The respondents’ age ranges from 18 to 74. The majority of samples range from age 25 to 44 (*n* = 274, 64.1%). We recruited an equal proportion of people from both genders (*n* = 213 for each). In terms of ethnicity, more than 70% of the respondents were White (*n* = 332, 77.8%), followed by Asian Pacific (*n* = 38, 8.9%), African American (*n* = 27, 6.3%) and Hispanic (*n* = 24, 5.6%). Approximately 74.2% of the respondents had some level of college education (*n* = 317), and 15.2% of the samples had professional degrees, master’s or doctorate (*n* = 65), while 10.5% of the samples have had a high school degree or less (*n* = 45). More than half of the sample has a fixed income less than $50,000 (*n* = 242, 56.7%), 26.9% earn $50,000 to less than $80,000, and approximately 16.4% have a yearly income of $80,000 to more than $100,000 (*n* = 70).

### Procedure

The online survey consisted of two sections. The first section of the questionnaire asked about respondents’ knowledge of the substandard and falsified medicines challenge, risk aversion, the ethicality of buying or selling fake medicines, subjective norms about the issue, risk perception, perceived benefit, attitudes, and purchase intention of purchasing SFMs. Demographic information includes age, gender, income, and educational background. Before answering the actual questions, the researchers informed the respondents: “The term ‘counterfeit’ is used to describe products that are deliberately mislabeled with respect to their identity and/or source. Counterfeiting can apply to both branded and generic products. It may include products that contain the wrong ingredients, without active ingredients, with insufficient quantities of ingredient(s), or with fake packaging.”

### Measurement reliability

All items were measured using a five-point Likert scale (1 = strongly disagree, 5 = strongly agree). The measures used for this study include knowledge of SFMs, perceived value, perceived risks, attitude toward counterfeit drugs, subjective norms about SFMs, ethical judgment, risk aversion, behavioral control, and purchase intention. All computed Cronbach’s alphas are reliable.

#### Knowledge of SFMs

The study used a three-item measure (adapted from Yoo and Donthu [57]) to assess respondents’ awareness of SFMs. The statements include: “I can recognize counterfeit medicines among other genuine brands,” “I am aware of counterfeit products,” and “Some characteristics of counterfeit medicine come to my mind quickly” (α = 0.73, M = 2.71).

#### Perceived risk

Five items were adapted and used to assess the risks participants associate with consuming SFMs (α = 0.92, M = 3.65) [37, 58].

#### Perceived value/benefits

A three-item adapted measure of perceived benefit [58] of consuming counterfeit medicines was also administered (α = 0.95, M = 1.78) and used as a covariate.

#### Attitude toward SFMs

Fourteen items asking about the respondents’ attitude toward SFMs were adapted from the literature [39, 46]. The items asked about participants' attitudes toward buying and selling SFM (α = 0.98, M = 1.60).

#### Subjective norm about SFMs

Seven items [33] were adapted and used to assess the variable asking how the respondents know would think of buying SFMs (α = 0.92, M = 2.07).

#### Ethical judgment

Five items assessing the respondents’ ethical judgments regarding buying and selling SFMs were used (α = 0.85, M = 3.94). Three questions were adopted from a previous study [59], and two additional researcher-generated items were added.

#### Risk aversion

Eight items were used to evaluate the respondents’ general risk aversion and aversion to SFMs (α = 0.78, M = 3.87) [46, 60].

#### Purchase intention

Seven items were used to assess the respondents’ likelihood of buying SFMs (α = 0.86, M = 1.80). The seven-item scale was adapted from Sweeney, Soutar, and Johnson [58] and Chakraborty et al. [37].

## Results

### Perceived risk, consumer attitude, and intent to consume SFMs

Our test of H1a found a negative relationship between perceived risk of SFMs and consumers’ overall attitudes toward such medicines (β = -0.59, B = -1.95, t(425) = -15.20, *p* < 0.001, *R*^2^ = 0.35, F(1, 425) = 230.98, *p* < 0.001). Risk perception explains 35 percent of the variance in consumers’ attitudes toward counterfeits. The relationship is such that a 100-point increase in risk perception is associated with a 25-point reduction in how favorable consumers’ views on counterfeits are.

H1b predicted a negative relationship between risk perception and SFMs purchase intentions. This hypothesis was also supported (β = -0.61, B = -0.15, t(425) = -15.97, *p* < 0.001, *R*^2^ = 0.38, F(1, 425) = 255.12, *p* < 0.001). Thus, risk perception explains 38 percent of the variance in consumers’ intention to purchase SFMs.

### Ethical judgment, attitude, risk perception, and purchase intention

Our analysis also found support for the hypothesis (H2a) that consumers’ ethical judgment about SFMs is inversely related to their overall attitude toward consuming such medicines (β = -0.45, B = -6.97, t(425) = -10.29, *p* < 0.001, *R*^2^ = 0.20, F(1, 425) = 105.86, *p* < 0.001). Ethical judgment explains 20 percent of the variance in attitudes toward counterfeits. Similarly, we found support for the predicted relationship (H2b) between ethical judgment and risk perception (β = 0.51, B = 2.43, t(425) = 12.29, *p* < 0.001, *R*^2^ = 0.26, F(1, 425) = 151.09, *p* < 0.001). Thus, higher risk perception is associated with consumers who view buying and selling SFMs as unethical. Ethics explains 26 percent of the variance in risk perceptions.

The results also support our hypothesis (H2c) regarding ethical judgments and purchase intention. Consumers who view SFMs as unethical are less intent on purchasing such medicines (β = -0.47, B = -0.54, t(425) = -11.05, *p* < 0.001, R^2^ = 0.22, F(1, 425) = 122.06, *p* < 0.001).

### Knowledge of counterfeit drugs, attitude, risk perception, and purchase intention

Contrary to H3a, we found a positive relationship between consumer knowledge and attitude toward SFMs (β = 0.32, B = 1.29, t(425) = 6.98, *p* < 0.001, *R*^2^ = 0.10, F(1, 425) = 48.68, *p* < 0.001). Thus, surprisingly, consumers who are more aware of the phenomenon of SFMs tend to have more favorable views on SFMs than those who claim not to be aware of the problem. A 100-point increase in consumers’ knowledge is associated with an approximately 32-point increase in favorable attitudes toward the issue. Knowledge explains 10 percent of the variance in consumers’ attitudes. Also, contrary to H3b, we found a negative relationship between knowledge and perceived risk of counterfeit drugs (β = -0.16, B = 0.19, t(425) = -3.26, *p* = 0.001, *R*^2^ = 0.02, F(1, 425) = 10.61, *p* < 0.001). Thus, surprisingly, consumers who are more aware of SFMs tend to associate the phenomenon with lower risks than those who claim not to be aware of the problem. A 100-point increase in consumers’ knowledge is linked to a 16-point reduction in the risks they associate with consuming SFMs. But this variable explains only two percent of the variance in risk perceptions.

H3c predicted a negative link between consumers’ knowledge and intentions to purchase or use SFMs. A significant relationship was found but in the reverse direction (β = 0.25, B = 0.07, t(425) = 5.22, *p* < 0.001, *R*^2^ = 0.06, F(1, 425) = 27.22, *p* < 0.001). Thus, contrary to our expectations, people aware of SFMs are more willing to purchase and consume such products than those who are not.

### Subjective norm, attitude, risk perception, and purchase intention

The study found support for H4a, which predicted a positive relationship between consumers’ subjective norm toward purchasing SFMs and their overall attitude toward the sale and consumption of counterfeit drugs (β = 0.60, B = 7.18, t(425) = 14.47, *p* < 0.001, *R*^2^ = 0.33, F(1, 425) = 208.23, *p* < 0.001). Thus, consumers who think their friends and loved ones will approve of them consuming SFMs tend to have an overall favorable attitude toward such medicines than people who think their loved ones will disapprove of such a practice. Subjective norm explains 33% of the variance in consumer attitudes. A 100-point increase in subjective norm is associated with a 60-point reduction in risk perception.

Additionally, the study found support for H4b, which predicted a negative relationship between consumers’ subjective norm toward purchasing SFMs and their risk perceptions (β = -0.60, B = -2.27, t(425) = -15.35, *p* < 0.001, *R*^2^ = 0.36, F(1, 425) = 235.62, *p* < 0.001). Thus, consumers who think their friends and loved ones will approve of them consuming SFMs tend to perceive lower risks than those who think their loved ones will disapprove of such a practice. The relationship between subjective norm and risk perception is such that, for example, a 100-point increase in subjective norm is associated with a 60-point reduction in risk perception.

H4c predicted a positive relationship between consumers’ subjective norm toward purchasing SFMs and their purchase intentions. This was supported (β = 0.58, B = , 0.53, t(425) = 14.61, *p* < 0.001, *R*^2^ = 0.33, F(1, 425) = 213.58, *p* < 0.001). Hence, consumers who think their friends and loved ones will disapprove of their decision to SFMs are more likely to say they do not intend to purchase such medicines. Moreover, subjective norm explains a third of the variance in consumers’ purchase intentions regarding fake medicines.

### Risk aversion, consumer attitude, risk perception, and purchase intention

Our hypothesis (H5a) regarding risk aversion and consumers’ attitude toward the purchase of SFMs was supported (β = -0.45, B = -7.64, t(425) = -10.45, *p* < 0.001, *R*^2^ = 0.20, F(1, 425) = 109.18, *p* =  < 0.001). This variable explains 20 percent of the variance in consumer attitudes toward purchasing SFMs. The relationship is such that a 100-point increase in aversion is linked with a 45-point decline in attitudes toward SFMs. H5b predicted a positive relationship between risk aversion and consumers’ risk associated with counterfeit medicine consumption. The analysis found support for this hypothesis (β = 0.49, B = 2.52, t(425) = 11.61, *p* < 0.001, *R*^2^ = 0.24, F(1, 425) = 134.78, *p* < 0.001). Risk aversion explains only 24 percent of the variance in the risk consumers associated with consuming SFMs. Our test of H5c also found support for the hypothesis that there is an inverse relationship between consumers’ risk aversion and the intentions to purchase SFMs (β = -0.50, B = -0.61, t(425) = -11.89, *p* < 0.001, *R*^2^ = 0.25, F(1, 425) = 141.26, *p* < 0.001).

### Income, attitude toward SFMs, risk perception, and purchase intention

To test our hypothesis (H6a) regarding consumers’ income level and their attitudes toward SFMs, we conducted a one-way ANOVA test. We found a significant difference among consumers of certain income groups (F(4, 422) = 2.41, *p* < 0.05). Additional post-hoc tests found that consumers who earn less than $20,000 had more favorable attitudes toward counterfeit drugs (m = 22.69, sd = 11.72) than those who earn between $20,000 and $50,000 (m = 19.18, sd = 9.36, *p* < 0.05). Also, the $20,000 and $50,000 (m = 19.18, sd = 9.36) income bracket group had less favorable views on SFMs that those earning $80,000 and $100,000 (m = 23.59, sd = 13.17, *p* < 0.05). We found no difference for the other income groups.

To test our hypothesis (H6b) regarding the income and SFMs risk perception, we conducted a one-way ANOVA. The analysis found no significant differences in risk perception (F(4,422) = 1.32, *p* > 0.05). Hypothesis 2c regarding the income and SFMs purchase intention also found an insignificant relationship between income levels and SFMs purchase intention (F(4, 422) = 1.23, *p* > 0.05).

### Age, attitude toward SFMs, risk perception, and purchase intention

A series of regression tests were conducted to address our research questions regarding age and the following outcomes: attitudes, risk perceptions, and purchase intentions. First, regarding RQ1a, consumers’ attitude toward SFMs were found to differ by age (β = -0.17, B = -1.44, t(425) = -3.44, *p* = 0.001, *R*^2^ = 0.03, F(1, 425) = 11.82, *p* = 0.001). Thus, older people tend not to like SFMs.

Second, regarding RQ1b, age was positively associated with SFMs risk perceptions (β = 0.21, B = 0.55, t(425) = 4.40, *p* < 0.001, *R*^2^ = 0.04, F(1, 425) = 19.34, *p* = 0.001). Thus, older people associate SFMs with higher risks than younger consumers do.

Our test regarding RQ1c, returned a significant negative association between consumers’ age and their intentions to purchase SFMs (β = -0.19, B = -0.12, t(425) = -4.02, *p* < 0.001, *R*^2^ = 0.04, F(1, 425) = 16.14, *p* = 0.001). That is, younger consumers are more likely to consume SFMs than older people.

### Overall model predicting consumer attitudes, risk perception, and behavior intention

We conducted a series of multiple regressions to test the combined effect of age, knowledge, ethical judgment, risk aversion, and subjective norm on risk perceptions, attitude toward counterfeit medicine consumption, and purchase intentions (R.Q. 2a – 2c). See Table 1 for how these predictors correlate to each other.

**Table 1:** Correlation matrix of all predictors

	Age	Knowledge	Subjective Norm	Ethical Judgment	Risk Aversion
Age					
r	1				
Sig					
Knowledge					
r	-.027	1			
Sig	.584				
Subjective Norm					
r	-.083	.125^b^	1		
Sig	.088	.010			
Ethical Judgment					
r	.103^a^	-.091	-.601^b^	1	
Sig	.034	.061	.000		
Risk Aversion					
r	.099^a^	-.163^b^	-.422^b^	.432^b^	1
Sig	.042	.001	.000	.000	

^a^Correlation is significant at the 0.05 level (2-tailed). *N* = 427

^b^Correlation is significant at the 0.01 level (2-tailed)

First, a multiple linear regression was calculated to predict consumer attitudes toward “counterfeit medicine” consumption based on their age, knowledge, ethical judgment, risk aversion, subjective norm, and perceived benefit. (Income does not significantly improve the model; we have, therefore, excluded it from the results for parsimony.) The overall model (Model 2) explains more than a two-thirds of the variance in consumer attitude toward SFMs (F(6, 420) = 162.30, *p* < 0.001, *R*^2^ = 0.70). As the standardized betas show in Table 2, controlling for all other factors, perceived benefit is positively associated with attitudes, and is the most significant predictor of consumer attitudes toward SFMs. This is followed by subjective norm, risk aversion, and self-reported knowledge of the substandard and falsified medicines problem. Age and consumers’ ethical judgments about buying or selling SFMs are not significant factors in the predictive model (*p* = 0.44 and 0.50, respectively).

**Table 2:** Summary of hierarchical regression analysis for variables predicting attitude toward counterfeit medicine consumption (*N* = 427)

Model		Unstandardized Coefficients		Standardized Coefficients	t	Sig
		B	S.E. B	β		
1	(Constant)	40.777	5.786		7.048	.000
	Age	-1.435	.417	-.165	-3.439	.001
	F = 11.82					.001
	*R*^2^ = .03					
2	(Constant)	10.691	4.610		2.319	.021
	Age	-.182	.238	-.021	-.766	.444
	Knowledge	.350	.114	.087	3.070	.002
	Subjective Norm	2.775	.447	.222	6.208	< .001
	Ethical Judgment	-.364	.543	-.023	-.670	.503
	Risk Aversion	-1.792	.526	-.106	-3.406	< .001
	Perceived Benefit	3.035	.163	.609	18.676	< .001
	F = 162.30					< .001
	*R*^2^ = .70					< .001

For subjective norm, a high score implies a belief that friends and loved ones will approve of them consuming SFMs. A high score on ethical judgment implies a belief that counterfeit medicine sale and consumption is unethical

Second, a multiple linear regression was calculated to predict consumers’ perception of the risks associated with counterfeit medicine consumption based on age, knowledge, ethical judgment, risk aversion, subjective norm, and perceived benefit of SFMs. The overall model (Model 2) explains more than half of the variance in the risk consumers associate with SFMs (F(6, 420) = 76.07, *p* < 0.001, *R*^2^ = 0.52). From Table 3, controlling for the other factors, the standardized coefficients show that subjective norm is the most significant predictor of the risk individuals associate with counterfeit medicine consumption. This was followed by perceived benefits, risk aversion, ethical judgment, and age. Self-reported knowledge does not significantly improve the model (*p* = 0.16).

**Table 3:** Summary of hierarchical regression analysis for variables predicting perceived counterfeit medicine risk (*N* = 427)

Model		Unstandardized Coefficients		Standardized Coefficients	t	Sig
		B	S.E. B	β		
1	(Constant)	10.617	1.747		6.078	.000
	Age	.554	.126	.209	4.398	.000
	F = 19.34					
	*R*^2^ = .04					
2	(Constant)	12.449	1.770		7.032	< .001
	Age	.264	.091	.100	2.895	.004
	Knowledge	.020	.044	.016	.448	.655
	Subjective Norm	-1.132	.172	-.297	-6.594	< .001
	Ethical Judgment	.614	.209	.129	2.941	.003
	Risk Aversion	1.000	.202	.194	4.952	< .001
	Perceived Benefit	-.441	.062	-.290	-7.059	< .001
	F = 76.07					< .001
	*R*^2^ = .52					< .001

Third, we run a multiple linear regression to predict consumers’ intention to purchase SFMs based on age, knowledge, ethical judgment, risk aversion, subjective norm, and perceived benefit. The overall model (Model 2) explains about 56 percent of the variance in the consumers’ intention to purchase SFMs (F(6, 420) = 87.31, *p* < 0.001, *R*^2^ = 0.56). As seen in Table 4, the standardized betas indicate that (controlling for all other factors), perceived benefit is the best predictor (with a positive association) of consumers’ purchase intention, followed by subjective norm, risk aversion, and age (in that order). Ethical judgment (*p* = 0.11) and knowledge (*p* = 0.11) has no significant effect after controlling for all the other predictors.

**Table 4:** Summary of hierarchical regression analysis for variables predicting SFMs purchase intentions (*N* = 427)

Model		Unstandardized Coefficients		Standardized Coefficients	t	Sig
		B	S.E. B	β		
1	(Constant)	3.478	.419		8.304	.000
	Age	-.121	.030	-.191	-4.017	.000
	F = 16.14					.000
	*R*^2^ = .04					
2	(Constant)	2.481	.407		6.090	< .001
	Age	-.046	.021	-.072	-2.173	.030
	Knowledge	.016	.010	.055	1.590	.113
	Subjective Norm	.239	.040	.263	6.059	< .001
	Ethical Judgment	-.077	.048	-.068	-1.605	.109
	Risk Aversion	-.247	.046	-.201	-5.309	< .001
	Perceived Benefit	.139	.014	.383	9.662	< .001
	F = 87.31					< .001
	*R*^2^ = .56					< .001

Finally, based on the theory of planned behavior’s proposition that subjective norms, attitudes, and perceptions influence individuals’ behavioral intentions [26, 27], the researchers estimated a predictive model for consumers’ intentions to patronize SFMs. The predictors include age, knowledge, subjective norm, ethical judgment, risk aversion, attitude, risk perception, and perceived benefit. The final model explains 65 percent of the variance in the consumers’ intention to purchase SFMs (F(8, 418) = 98.98, *p* < 0.001, *R*^2^ = 0.65). Age (*p* = 0.11), knowledge (*p* = 0.69), and ethical judgment (*p* = 0.31) have no significant effects on individuals’ intention to consume SFMs. Interestingly, also, perceived value/benefit does not have a significant effect on intentions (*p* = 0.51). Controlling for all other factors, consumers attitudes is the largest predictor of their behavioral intentions (β = 0.51, *p* < 0.001), followed by perceived risks (β = -0.13, *p* = 0.002), risk aversion (β = -0.12, *p* < 0.001) and subjective norms (β = -0.12, *p* < 0.009).

## Discussion

To the best of our knowledge, this study is the first to examine the social and psychological predictors of consumer attitudes toward SFMs in the United States. Our results are therefore useful for further inquiry and practice. The research is based on the view that beyond product packaging—which is an unreliable marker of authenticity [25]—social, psychological, and normative considerations can help understand how consumers relate to counterfeit products—in this case, medicines. As a first step toward understanding how consumers think about counterfeit drugs, this research examined how factors such as knowledge, income, age, ethical judgment, risk aversion, subjective norm (or normative susceptibility) help explain (a) what consumers think about SFMs, (b) the risks they associate with consuming such medication, as well as (c) their intentions to purchase.

Based on existing research [29, 31], one would expect that having prior knowledge of SFMs will valence people’s attitudes toward the problem. However, our hypothesis testing suggests that self-reported knowledge of the SFMs challenge is associated with favorable consumer attitudes and purchase intentions. Three possible reasons might explain this result. First, as earlier studies [39, 42] found in non-pharmaceutical contexts, being aware of, knowing about, or having consumed counterfeit products in the past, is not necessarily associated with unfavorable attitudes toward such products. It is, therefore, plausible that for the consumers, statements such as “I can recognize counterfeit medicines among other genuine brands,” “I am aware of counterfeit medicines,” and “Some characteristics of counterfeit medicines come to my mind quickly” serve as proxies for personal experience with SFMs. Thus, the finding that self-reported knowledge is positively associated with attitude toward counterfeit drugs and purchase intentions (but negatively associated with risk perception) aligns with earlier studies on individuals past counterfeit products consumption and attitudes [42, 46]. A second explanation for these results comes from the risk perception and decision science literature: familiarity and habituation. When people see risky phenomena or hazards as familiar or known (as opposed to novel), they discount how dangerous those phenomena are despite that the objective level of the risk remains the same (see Slovic [61]).

Further, risk perception mediates consumers’ evaluations of counterfeit products [37]. In other words, being aware of SFMs may not lead to unfavorable attitudes if we conceptualize knowledge as familiarity. Thus, to “know something” is to be “familiar with it,” and familiarity has a discounting effect on risk perception. A third possible explanation is that consumer knowledge serves as a proxy for self-efficacy, which attenuates consumer attitudes [33, 46]. These hypotheses are all fertile grounds for further testing.

The study also reports on two demographic variables: income and age. Contrary to studies [39, 41] that link having a low income to favorable attitudes and SFM purchase intentions, our results suggest that the relationship between income and how consumers feel about substandard and falsified medicines is mixed, and may not be linear. While consumers who earn less than $20,000 had more favorable attitudes toward counterfeit drugs than those who make between $20,000 and $50,000, those who make income higher than $50,000 are no different from all other groups. This finding aligns with Tom et al.’s [41] — but contrary to Bian and Moutinho’s [42]— results on consumer attitudes toward counterfeit products in general. This study also found younger consumers to be more risk-tolerant and have favorable attitudes toward SFMs. This finding, coupled with high internet usage among younger people, may make them more susceptible to illicit online pharmacies [62].

Also, corroborating results from earlier studies from other counterfeit product categories [39, 40], this study supports the hypothesized link between risk perception, attitudes toward SFMs, and purchase intentions. Thus, when consumers see counterfeit drugs as risky in terms of long-term health implications, costs, and efficacy, they are less likely to express intent to patronize such medicines. It suggests that awareness creation that focuses on personal risks and negative cues [36, 37] could enhance consumer decision-making about counterfeit drugs. While this study does not test for the mediation effect of risk perception on attitudes and consumers’ intention to purchase SFMs, given the pattern of results obtained in this exploratory study, it is reasonable to expect some form of mediation or moderation effects. Regarding perceived risk versus benefit of SFMs: It is plausible that even if consumers associate risks with “counterfeit” medicines, it might be worth the risk for them if they believe the benefit outweighs the cost. However, as we illustrate, the perceived benefit/value of SFMs does not significantly affect consumption intent after controlling for risk perception, attitude, and subjective norm.

In summary, in line with the general literature on counterfeit products, consumer knowledge (albeit in an unexpected direction), people’s expectations about what will impress their significant others, their ethical judgments about selling and consuming counterfeits, and their risk-aversion are associated with their judgment and decision-making about SFMs. While subjective norm/normative susceptibility and perceived benefits are the most significant predictors of consumer attitudes, risk perceptions, and purchase intentions, these factors combined explain 52 to 70 percent of the variance in the specified outcomes. Despite contributing to our understanding of individuals’ attitudes toward fake medicines, this study acknowledges that consumers cannot always tell which drugs are counterfeit and which ones are not, given the sophisticated nature of packaging used in SFMs. Thus, given that packaging characteristics are not reliable markers of authenticity [1, 6, 25], knowing the factors that make consumers more receptive to counterfeit medicine consumption is essential for advocacy and public education.

Currently ongoing efforts include Alliance for Safe Online Pharmacies’ Buy SafeRx; U.S. Food and Drug Administration’s Know Your Source, Filled with Empty Promises and BeSafeRx; and Pfizer’s Fight the Fakes [63]. Like studies on counterfeiting in other non-pharmaceutical consumption contexts (e.g., Michaelidou and Christodoulides [45]), beyond simply seeking to raise awareness, these results have lessons for designing demand-side strategies that combat the SFM concern. Beyond these informational efforts, policymakers, advocates, and pharmaceutical firms need campaigns that discourage the consumption of SFMs by appealing to individuals’ desire to impress their significant others, risk aversion, and risk perception. Here, using a social desirability tactic that highlights how consuming SFMs can hurt one’s social standing, as well as emphasizing the health risk of consuming SFMs are demand-side strategies worth exploring. Their attitudes can also be shaped by appealing to their belief in a fair and equitable life.

### Limitations of the study and future studies

The most important limitation of the study derives from its exploratory nature. Based on our comprehensive literature review, this research is the first to examine the social and individual-level factors that underlie consumers’ attitudes toward SFMs. Future studies should build on the analysis to include, for example, mediation or moderation tests. For instance, while the study examines the relationship between risk aversion, knowledge, ethical judgments, subjective norms on attitudes, risk perception, and purchase intentions, it does not assess the complex relationship between these predictors. Subsequent studies could assess whether ethical judgments, risk aversion, and subjective norms mediate or moderate the effect of knowledge of consumer attitudes and purchase intentions. Besides, considering this study’s public health focus, we purposefully did not explore the legal dimensions of the “counterfeit medicines” challenge. In addition, this work does not focus so much on *where* individuals access SFMs. However, future studies would benefit from distinguishing between different avenues of SFM trafficking and access (e.g., illegal street markets, online, legitimate pharmacies, clinics, and hospitals). Besides, given that our analysis found a link between risk perception and consumer attitudes, risk perceptions might mediate the effect of knowledge, ethics, and people’s intrinsic need to engage in behaviors approved by people who are important to them. These results present another avenue for follow-up studies. These findings suggest interesting direction pharmaceutical firms, regulatory organizations, and consumer safety advocacy groups can explore in their public education and brand reputation protection campaigns. For example, regarding the link between risk perception and attitudes, pharmaceutical brands and safety advocates would benefit from using message cues that highlight risks, people’s need to be affirmed by their significant others, and the need for safety (and risk aversion). But, concerning creating awareness about the problem, care should be taken to not frame the issue in ways that enhance a false sense of self-efficacy. Plus, based on evidence from the risk psychology literature, we recommend framing the problem in ways that make the “novelty” (as opposed to the “familiarity”) of the problem salient. These recommendations also provide fertile grounds for further empirical testing.

## Supplementary Information

**Additional file 1.**

## Data Availability

The data reported in this study was part of a larger project. The dataset is currently not publicly available because analyses for separate articles are ongoing, but the corresponding author will make it available on reasonable request.

## Abbreviations

EAASM: European Alliance for Access to Safe Medicines
OECD: Organization for Economic Co-operation and Development
SFMs: Substandard and falsified medicines
SSFFC: Substandard, spurious, falsely-labeled, falsified, and counterfeits
WHO: World Health Organization

## References

[1] Bate R, Nugent R (2008). The deadly world of fake drugs. Foreign Policy.

[2] Organization for Economic Co-operation and Development (OECD). Trade in counterfeit pharmaceutical products. 2020. 10.1787/a7c7e054-en.

[3] O’Hagan A, Garlington A (2018). Counterfeit drugs and the online pharmaceutical trade, a threat to public safety. Forensic Res Criminol Int J.

[4] World Economic Forum. The global risks report. 11th ed. 2016. Available from: http://www3.weforum.org/docs/GRR/WEF_GRR16.pdf.

[5] Ghinea N, Lipworth W, Day R, Hill A, Dore GJ, Danta M (2017). Importation of generic hepatitis C therapies: bridging the gap between price and access in high-income countries. The Lancet.

[6] Kelesidis T, Kelesidis I, Rafailidis PI, Falagas ME (2007). Counterfeit or substandard antimicrobial drugs: a review of the scientific evidence. J Antimicrob Chemother.

[7] Alfadl AA, Ibrahim MIM, Hassali MA (2012). Consumer behaviour towards counterfeit drugs in a developing country. J Pharm Health Serv.

[8] McManus D, Naughton BD (2020). A systematic review of substandard, falsified, unlicensed and unregistered medicine sampling studies: a focus on context, prevalence, and quality. BMJ Glob Health.

[9] Wieland S. Counterfeit drugs and the supply chain into Africa: How Kenya could apply Rwanda’s supply chain model. 2020. Available from: https://www.academia.edu/9425341/COUNTERFEIT_DRUGS_AND_THE_SUPPLY_CHAIN_INTO_AFRICA_How_Kenya_could_apply_Rwandas_supply_chain_model?auto=download.

[10] World Health Organization 2018 Substandard, spurious, falsely labelled, falsified and counterfeit (SSFFC) medical products fact sheet Available at: http://www.who.int/mediacentre/factsheets/fs275/en/

[11] Rahman MS, Yoshida N, Tsuboi H, Tomizu N, Endo J, Miyu O (2018). Kimura, K The health consequences of falsified medicines-a study of the published literature. Trop Med Int Health.

[12] Miller HI, Winegarden W. Fraud in your pill bottle: The unacceptable cost of counterfeit medicines. Center for Medical Economics and Innovation Issue Brief. Pacific Research Institute; 2020.

[13] Gurney B, Amundson G, Boumediene SL (2017). Ways to battle the $431 billion fake pharmaceutical industry. Rev Business Finance Stud.

[14] Mansfield K, Wong P (2015). Combating counterfeit medicines with technology: a novel remedy to an increasing problem. Pharm Process.

[15] Hamilton WL, Doyle C, Halliwell-Ewen M, Lambert G (2016). Public health interventions to protect against falsified medicines: a systematic review of international, national and local policies. Health Policy and Plan.

[16] Maniace L. How do I know if an online pharmacy is legitimate? Consumer Reports News. 2011. Available at: http://www.consumerreports.org/cro/news/2011/10/save-money-by-ordering-drugs-from-canada-not-so-fast/index.htm

[17] National Association of Boards of Pharmacy. Internet drug outlet identification program progress report for state and federal regulators. 2017. Available at: https://nabp.pharmacy/wp-content/uploads/2016/08/Internet-Drug-Outlet-Report-August-2017.pdf

[18] Vida RG, Fittler A, Mikulka I, Ábrahám E, Sándor V, Kilár F, Botz L (2017). Availability and quality of illegitimate somatropin products obtained from the Internet. Int J Clin Pharm.

[19] Alliance for Safe Online Pharmacies. Key data about Illegal online drug sellers and counterfeit medicines. 2017. Available at: https://buysaferx.pharmacy//wp-content/uploads/2017/10/factsheet_keydata-1.pdf.

[20] Fittler A, Vida RG, Káplár M, Botz L (2018). Consumers turning to the internet pharmacy market: cross-sectional study on the frequency and attitudes of Hungarian patients purchasing medications online. J Med Internet Res.

[21] Lybecker KM (2007). Rx Roulette: combatting counterfeit pharmaceuticals in developing nations. Manag Decis Econ.

[22] Oliver A, Mossialos E (2004). Equity of access to health care: outlining the foundations for action. J Epidemiol Community Health.

[23] Park M, Ahn S (2012). Quantitative analysis of sildenafil and tadalafil in various fake drugs recently distributed in Korea. Forensic Sci.

[24] Wechsler J (2012). Campaign mounts to curb counterfeit drugs: manufacturers and regulators struggle to control phony versions of crucial medicines. BioPharm Int.

[25] Po ALW (2001). Too much, too little, or none at all: dealing with substandard and fake drugs. Lancet.

[26] Ajzen I (1991). The theory of planned behaviour. Organ Behav Hum Decis Process.

[27] Ajzen I, Fishbein M (2004). Questions raised by a reasoned action approach: Comment on Ogden (2003). Health Psychol.

[28] Cordell VV, Wongtada N, Kieschnick RL (1996). Counterfeit purchase intentions: role of lawfulness attitudes and product traits as determinants. J Bus Res.

[29] Khalid M, Rahman SU (2015). Word of mouth, perceived risk and emotions, explaining consumers’ counterfeit products purchase intentions in a developing country: Implications for local and international original brands. Adv Business Rel Sci Res.

[30] Lee KS, Yee SM, Zaidi STR, Patel RP, Yang Q, Al-Worafi YM, Ming LC (2017). Combating sale of counterfeit and falsified medicines online: a losing battle. Front Pharmacol.

[31] Swoboda B, Pennemann K, Taube M. Purchasing the Counterfeit: Antecedences and Consequences from Culturally diverse Countries. In: Foscht T, Morschett D, Rudolph T, Schnedlitz P, Schramm-Klein H, Swoboda B, editors. European Retail Research. Wiesbaden: Springer; 2014;27(1):23–41.

[32] Wilcock AE, Boys KA (2014). Reduce product counterfeiting: An integrated approach. Bus Horiz.

[33] Armitage CJ, Armitage CJ, Conner M, Loach J, Willetts D (1999). Different perceptions of control: applying an extended theory of planned behavior to legal and illegal drug use. Basic Appl Soc Psychol.

[34] Augusto de Matos C, Trindade Ituassu C, Vargas Rossi CA (2007). Consumer attitudes toward counterfeits: a review and extension. J Consum Mark.

[35] Basu M, Basu S, Lee JK. Factors influencing consumers intention to buy counterfeit products. Glob J Manag Bus Res. 2015;15(6):23–35. https://hdl.handle.net/1805/23648.

[36] Casola L, Kemp S, Mackenzie A (2009). Consumer decisions in the black market for stolen or counterfeit goods. J Econ Psychol.

[37] Chakraborty G, Allred A, Sukhdial AS, Bristol T. Use of negative cues to reduce demand for counterfeit products. In: Brucks M, MacInnis DJ, editors. NA-Advances in consumer research. Provo (UT): Association for Consumer Research; 1997;24:345–49.

[38] Commuri S (2009). The impact of counterfeiting on genuine-item consumers’ brand relationships. J Mark.

[39] Hoon Ang S, Sim Cheng P, Lim EA, Kuan TS (2001). Spot the difference: consumer responses toward counterfeits. J Consum Mark.

[40] Penz E, Stöttinger B (2005). Forget the ‘real’ thing – Take the copy! An explanatory model for the volitional purchase of counterfeit products. Adv Consum Res.

[41] Tom G, Garibaldi B, Zeng Y, Pilcher J (1998). Consumer demand for counterfeit goods. Psychol Mark.

[42] Bian X, Moutinho L (2009). An investigation of determinants of counterfeit purchase consideration. J Bus Res.

[43] Gentry JW, Putrevu S, Shultz CJ (2006). The effects of counterfeiting on consumer research. J Consum Behav.

[44] Huang JH, Lee BCY, Ho SH (2004). Consumer attitudes towards gray market goods. Int Market Rev.

[45] Michaelidou N, Christodoulides G (2011). Antecedents of attitude and intention towards counterfeit symbolic and experiential products. J Mark Manag.

[46] Chiu W, Leng HK (2015). Is that a Nike? The purchase of counterfeit sporting goods through the lens of the theory of planned behavior. Choregia Sport Manag Int J.

[47] Finlay KA, Trafimow D, Moroi E (1999). The importance of subjective norms on intentions to perform health behaviors. J Appl Soc Psychol.

[48] Kim H, Karpova E (2010). Consumer attitudes toward fashion counterfeits: Application of the theory of planned behavior. Cloth Text Res J.

[49] Wee CH, Ta SJ, Cheok KH (1995). Non-price determinants of intention to purchase counterfeit goods: An exploratory study. Int Mark Rev.

[50] Forman RF, Block LG (2006). The marketing of opioid medications without prescription over the internet. J Public Policy Mark.

[51] Littlejohn C, Baldacchino A, Schifano F, Deluca P. Internet pharmacies and online prescription drug sales: a cross-sectional study. Drugs Educ Prev Policy. 2005;12:75–80.

[52] Arceneaux K (2012). Cognitive biases and the strength of political arguments. Am J Pol Sci.

[53] Berinsky AJ, Margolis MF, Sances MW (2014). Separating the shirkers from the workers? Making sure respondents pay attention on self-administered surveys. Am J Pol Sci.

[54] Berinsky AJ, Huber GA, Lenz GS (2012). Evaluating online labor markets for experimental research: Amazon. Com’s Mechanical Turk Polit Anal.

[55] Huff C, Tingley D (2015). “Who are these people?” Evaluating the demographic characteristics and political preferences of MTurk survey respondents. Res Polit.

[56] Clifford S, Jewell RM, Waggoner PD (2015). Are samples drawn from Mechanical Turk valid for research on political ideology?. Res Polit.

[57] Yoo B, Donthu N (2001). Developing and validating a multidimensional consumer-based brand equity scale. J Bus Res.

[58] Sweeney JC, Soutar GN, Johnson LW (1999). The role of perceived risk in the quality-value relationship: a study in a retail environment. J Retail.

[59] Norum PS, Cuno A (2011). Analysis of the demand for counterfeit goods. J Fash Mark Manag.

[60] Raju PS (1980). Optimum stimulation level: Its relationship to personality, demographics, and exploratory behavior. J Consum Res.

[61] Slovic PE 2010 The feeling of risk Earthscan publications

[62] Niemz K, Griffiths M, Banyard P (2005). Prevalence of pathological Internet use among university students and correlations with self-esteem, the General Health Questionnaire (GHQ), and disinhibition. Cyber Psychol Behav.

[63] Ofori-Parku SS (2022). Fighting the global counterfeit medicines challenge: a consumer-facing communication strategy in the US is an imperative. J Glob Health.

