# Supplementary material for: I (Don’t) want to consume counterfeit medicines: exploratory study on the antecedents of consumer attitudes toward counterfeit medicines
Source: BMC Public Health. 2022 Jun 1;22:1094. doi: 10.1186/s12889-022-13529-7 (PMC9158175; doi:10.1186/s12889-022-13529-7)
Supplement: Supplementary file 1 — Additional file 1. [file 12889_2022_13529_MOESM1_ESM.doc]

**Appendix: Scale Items Used for Study**

All items were anchored on: “To what extent do you agree or disagree with the following statements about counterfeit?” (1= strongly disagree, 5 = strongly agree)

**Self-reported Knowledge**

- I can recognize counterfeit medicines among other genuine brands.
- I am aware of counterfeit medicines.
- Some characteristics of counterfeit medicines come to my mind quickly.

**Subjective Norm**

- People who are important to me would think I should not use counterfeit medicines
- People who are important to me would disapprove of my using counterfeit medicines.
- People who are important to me wouldn't want me to use counterfeit medicines.
- If I purchase counterfeit medicines, most of the people who are important to me will disapprove.
- The people I care about will look down on me if I purchase counterfeit medicines.
- My family members will think it is not okay to purchase medicines.
- My friends would think that buying counterfeit medicines is wrong.

**Ethical Judgement**

- People who buy counterfeit medicines are committing a crime.
- People who sell counterfeit medicines are committing a crime.
- Counterfeit medicines do not hurt the U.S. economy.
- Counterfeit medicines hurt the companies that manufacture the legitimate product.
- Producing counterfeit medicines is immoral.

**Risk Aversion**

- I never buy something I don’t know about (at the risk of making a mistake).
- If I buy medicines, I buy only well-established brands.
- When I buy a drug, I feel it is safer to buy a drug from a brand I am familiar with.
- When I buy a drug, I like to try the most unusual item even if I am not sure I would like it.
- I don't like to take risks.
- Compared to most people I know I like to live life on the edge.
- I have no desire to take unnecessary chances on things.
- Compared to most people I know, I like to gamble on things.

**Attitude toward Counterfeit Medicines**

- Buying counterfeit medicines would make me feel good.
- I would love it when counterfeits are available for medicines I need.
- For most medicines, the best buy is usually counterfeit medicines.
- In general, counterfeit medicines are of similar quality to genuine ones.
- Considering value for the money, I prefer counterfeit medicines to genuine ones.
- If I buy counterfeit medicines, I will feel that I am getting a good deal.
- Generally speaking, buying counterfeit medicines is a better choice.
- I would enjoy shopping for counterfeit medicines.
- Buying counterfeit medicines generally benefits the consumer.
- There is nothing wrong with purchasing counterfeit medicines.
- There is nothing wrong with selling counterfeit medicines.
- Generally speaking, buying counterfeit medicines is a better choice.
- Considering price, I would prefer buying counterfeit medicines to the originals.
- For most medicines, the best buy is counterfeits.

**Risk Perception**

- There is a chance that there will be something wrong with counterfeit medicines.
- There is a chance that counterfeit medicines will not work properly.
- There is a chance that buying a counterfeit medicine will make me lose money because it won’t work as expected.
- Counterfeit medicines are extremely risky (vs. not risky) in terms of their performance.
- Counterfeit medicines are extremely risky in terms of their long-term costs.

**Perceived Value/Benefit**

- I feel counterfeit medicines are good value for money.
- I believe at good price, counterfeit medicines are a good buy.
- I think counterfeit medicines are a good buy.

**Counterfeit Medicines Purchase Intention**

- I wouldn’t mind purchasing counterfeit medicines.
- Given a choice, my friends will choose counterfeit medicines.
- There is a strong likelihood that I will buy counterfeit medicines.
- I wouldn't mind recommending counterfeit medicines to my friends.
- Given a choice, I am willing to buy counterfeit medicine.
- I will never consider buying counterfeit medicines.
- The probability that I will consider buying counterfeit medicine is low.
